# Timing and Support Matter: Caregiving Demands at the Intersection of Stress Process and Life Course Perspectives

**DOI:** 10.1093/geroni/igab046.399

**Published:** 2021-12-17

**Authors:** Yeonjung Lee, Alex Bierman, Margaret Penning

**Affiliations:** 1 University of Calgary, Calgary, Alberta, Canada; 2 University of Calgary, University of Calgary, Alberta, Canada; 3 University of Victoria, Victoria, British Columbia, Canada

## Abstract

Extensive research documents the outcomes of family caregiving. However, perspectives differ, with some suggesting that caregiving provides psychological rewards and others suggesting that the stress of caregiving carries psychological costs. We argue that both of these perspectives are correct, but their applicability will differ based on the timing of caregiving and the availability of social support. A life course perspective suggests that the timing of a stressor in the life course will create variations in its mental health impacts, whereas a stress process perspective suggests that the consequences of a given stressor for mental health will vary based on the availability of social support. A synthesis of these two perspectives then suggests that social support will act as stress buffer differently depending on the age of caregiver. To examine these questions, we use a subsample of respondents who reported caregiving (N=20,441) in the 1st wave of the Canadian Longitudinal Study on Aging. Analyses provide evidence of different outcomes of caregiving, according to both the timing of caregiving and the availability of support. In particular, a high level of caregiving demands are associated with greater depression and lower life satisfaction. Social support inhibits both associations, and the association between high demands and life satisfaction is stronger in older caregivers. Social support does not buffer high caregiving demands more strongly at older ages, though, showing two distinct process. Demanding caregiving appears particularly detrimental for psychological well-being as people age, and the efficacy of social support resources do not increase to compensate.

